# The density of tumor-infiltrating lymphocytes and prognosis in resectable hepatocellular carcinoma: a two-phase study

**DOI:** 10.18632/aging.202710

**Published:** 2021-03-19

**Authors:** Fengwei Gao, Kunlin Xie, Qiwen Xiang, Yan Qin, Panyu Chen, Haifeng Wan, Yang Deng, Jiwei Huang, Hong Wu

**Affiliations:** 1 Department of Liver Surgery and Liver Transplantation, West China Hospital, Sichuan University, Chengdu, China; 2 Operation Room of West China Hospital, West China School of Nursing, Sichuan University, Chengdu, China; 3 Department of Surgery, Ruijin Hospital, Shanghai Jiao Tong University School of Medicine, Shanghai, China

**Keywords:** hepatocellular carcinoma, tumor-infiltrating lymphocytes, prognosis

## Abstract

Aim: Previous studies have focused on the subpopulations of tumor-infiltrating lymphocytes (TILs) in tumors. This study focuses only on the concentration of TILs in the tumor irrespective of type and elucidates its prognostic value.

Methods: We used 315 HCC patients as the discovery phase and another 343 HCC patients as the validation phase. By following the standardized guideline, density of TILs were categorized into low (TILs < 10%), intermediate (10% ≦ TILs < 50%), and high (TILs ≧ 50%) levels. Associations of TILs with prognostic, immune-related, and genetic variables were examined.

Results: We observed a dose-response relation of TILs with overall survival (intermediate: HR, 0.58; 95% confidence interval (CI), 0.36-0.93; high: HR, 0.37; 95% CI, 0.15-0.93) and disease-free survival (intermediate: HR, 0.35; 95% CI, 0.22-0.58; high: HR, 0.23; 95% CI, 0.09-0.58). The prognostic value of TILs was validated in the TCGA set. Mutation burden or the number of neoantigens were not associated with TILs intensity. However, hepatitis B or C virus infection patients had higher TILs intensity in the para-tumor tissue.

Conclusions: The TILs intensity was associated with patients' survival. If confirmed, this would suggest that clinical routine assessment of TILs could provide prognostic information in HCC.

## INTRODUCTION

The failure of immune surveillance to prevent the dissemination of tumor cells is closely associated with the disease-free survival (DFS) of patients with hepatocellular carcinoma (HCC). Cancer immunotherapies were seen to have considerable potential in controlling or eliminating minimal residual disease, consequently preventing recurrence and disease progression [1, 2]. Recent studies have demonstrated that pre-existing tumor-infiltrating lymphocytes (TILs) are important for the activity of immunotherapies including checkpoint blockade [3]. The presence of TILs is a sign of an immune-active tumor microenvironment, which may help identify patients who are more likely to benefit from immunotherapies.

The composition of TILs varies among tumor types, and the degree of TILs diverges from patient to patient [4]. TILs are a heterogeneous group comprised not only of effector T cells but also of regulatory T cells (Tregs), natural killer (NK) cells, macrophages, dendritic cells, myeloid-derived suppressor cells (MDSCs), and other immune cell types [5]. For HCC, emerging evidence has associated subsets of TILs with tumor progression, with either a pro- or anti-tumor activity. For instance, Tregs, M2 macrophages, and MDSCs have been reported to play central roles in dampening the anti-tumor immunity in HCC [6]. The presence of these cell types was also linked with inferior prognosis [7]. In contrast, the anti-tumorigenic subset, including CD8+ T cells, NK cells, T helper 1 cells, T helper 2 cells, and M1 macrophages, has been associated with a good prognosis [8]. Besides, the analysis of tumor-infiltrating B cells in HCC has yielded inclusive results, with both positive and negative prognostic associations being reported [9].

Given the critical role of TILs in shaping tumor immune microenvironment, more detailed knowledge of TILs will be essential for the development of novel immune-related approaches in HCC. The purpose of our study was to evaluate the prognostic significance of TILs in HCC. Moreover, we aimed to identify factors that might determine the intensity of TILs. To achieve this, we assessed the prognostic significance of TILs using data from 316 HCC patients in a discovery phase; another 370 HCC patients from The Cancer Genome Atlas (TCGA) were used for validation. Moreover, we determined whether the total mutation burden, neoantigen, and virus infection were associated with the intensity of TILs.

## RESULTS

### Characteristics of the study population and TILs distribution

The WCH set included 316 patients, and the TCGA set included 370 patients. The characteristics of our study population were presented in Supplementary Table 1. Compared to the WCH set, the TCGA set contained a significantly larger proportion of female participants. A statistically significant difference was observed regarding age, race, risk factor, and Ishak score between the two sets (*P* < 0.001). Specifically, patients from the WCH set tended to be younger and had higher Ishak scores, and more patients from the WCH set than from the TCGA set were Asian and positive for HBV infection. For 1 patient in the WCH set and 27 patients in the TCGA set, TILs could not be assessed due to tissue quality (Supplementary Figure 1). We found that 11% of tumors, in both the WCH set and the TCGA set, had high TILs (TILs ≧ 50%); 33% and 36% of tumors in the WCH set and the TCGA set had intermediate TILs (10% ≦ TILs < 50%), respectively; while 56% and 53% of tumors in the WCH set and the TCGA set had low TILs (TILs < 10%), respectively. We found that compared to the tumors with low TILs, those with high TILs had higher CD8, PD-1, PD-L1 on immune cells, and OX40 expression (Supplementary Table 2).

### Prognostic value of TILs

**Figure 1:**
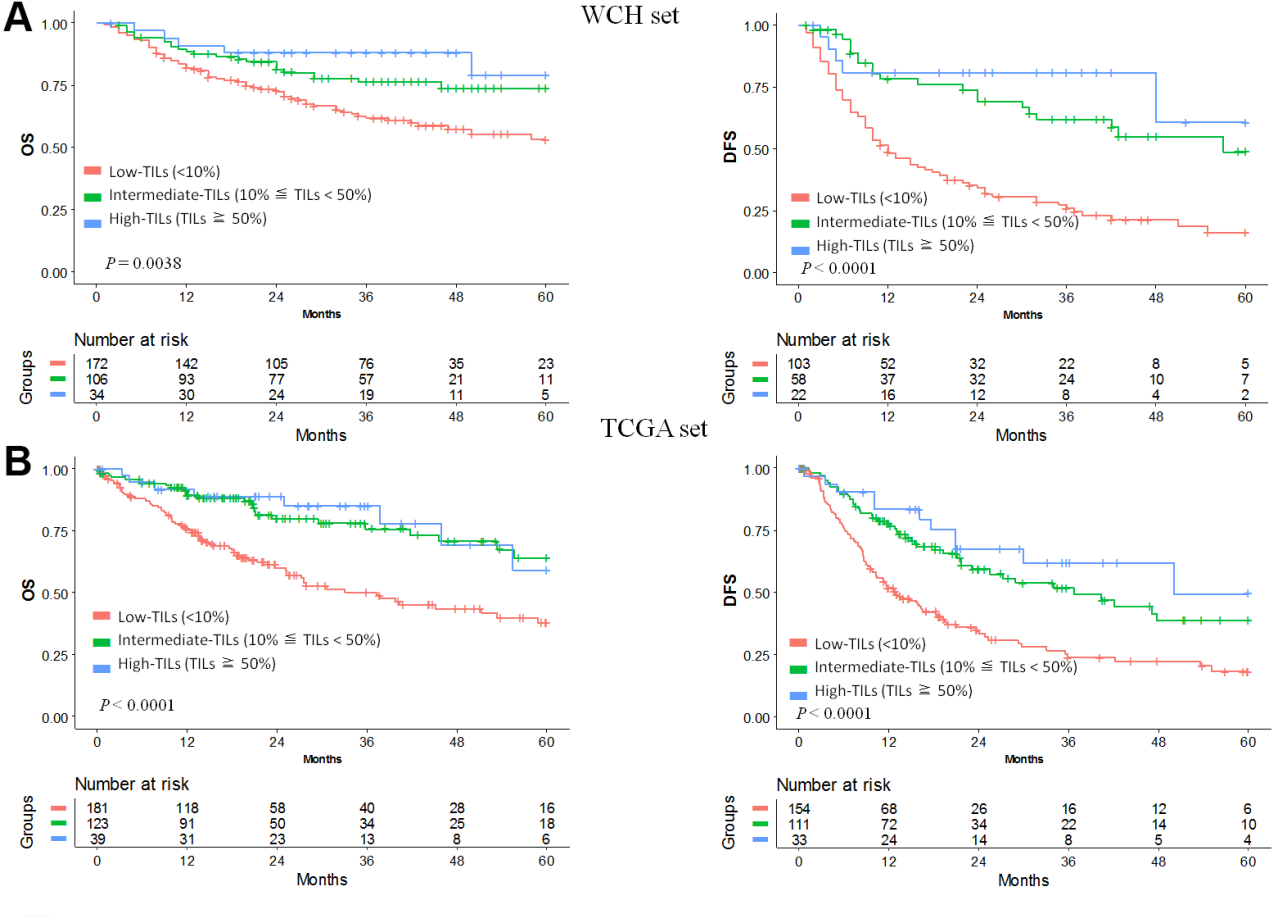
**Prognostic value of TILs in hepatocellular carcinoma.** Kaplan-Meier curves of estimated overall survival and disease-free survival in the WCH set (**A**) and the TCGA set (**B**). Patients without survival information were excluded from the analysis.

In the WCH set, univariable analysis and survival curves showed that the high- and intermediate-TILs groups had better OS than the low-TILs group (Figure 1A, Supplementary Table 3). The 5-year OS rate of the high-TILs group (79%; 95% CI, 62%-100%) was longer than that of the intermediate-TILs group (74%; 95% CI, 64%-84%) and the low-TILs group (53%; 95% CI, 44%-64%). Similarly, the 5-year DFS of the high-TILs group (61%; 95% CI, 33%-100%) was longer than that of the intermediate-TILs group (49%; 95% CI, 34%-70%) and the low-TILs group (16%; 95% CI, 9%-28%). After adjustment for age, sex, tumor grade and stage, our results confirmed that patients in the high- and intermediate-TILs group had better OS (intermediate: HR, 0.58; 95% CI, 0.36-0.93; high: HR, 0.37; 95% CI, 0.15-0.93; *P*_trend_, 4.25 × 10^-3^) and DFS (intermediate: HR, 0.35; 95% CI, 0.22-0.58; high: HR, 0.23; 95% CI, 0.09-0.58; *P*_trend_, 3.60 × 10^-6^) than patients in the low-TILs group (Table 1).

**Table 1:** Mortality risks by intensity of TILs for overall survival and disease-free survival.

**Group**	**TILs intensity**	**Overall survival**				**Disease-free survival**		
		**No. of** **cases/total**	**Adjusted** **HR** ^a^	***P***		**No. of** **cases/total**	**Adjusted** **HR** ^a^	***P***
**Discovery phase (WCH set)**								
Low	TILs < 10%	67/172	1 (reference)			80/103	1 (reference)	
Intermediate	10% ≦ TILs <50%	24/106	0.58 (0.36-0.93)	0.02		21/58	0.35 (0.22-0.58)	4.59 × 10^-5^
High	TILs ≧ 50%	5/34	0.37 (0.15-0.93)	0.04		5/22	0.23 (0.09-0.58)	1.83 × 10^-3^
***P*** for trend				4.25 × 10^-3^				3.60 × 10^-6^
**Validation phase (TCGA set)**								
Low	TILs < 10%	81/181	1 (reference)			100/154	1 (reference)	
Intermediate	10% ≦ TILs <50%	30/123	0.42 (0.28-0.66)	1.13 × 10^-4^		50/111	0.52(0.37-0.74)	2.78 × 10^-4^
High	TILs ≧ 50%	8/39	0.41 (0.19-0.86)	0.02		11/33	0.33 (0.17-0.62)	6.09 × 10^-4^
***P*** for trend				1.08 × 10^-4^				8.25 × 10^-6^

^a^Adjusted for age, sex, tumor stage and tumor grade.

We used the TCGA hepatocellular carcinoma cohort to validate the prognostic value of TILs. In line with results in the WCH set, patients in the low-TILs group had the worst 5-year OS and DFS (Figure 1B). The HR of risk for OS (intermediate: HR, 0.42; 95% CI, 0.28-0.66; high: HR, 0.41; 95% CI, 0.19-0.86; *P*_trend_, 1.08 × 10^-4^) and DFS (intermediate: HR, 0.52; 95% CI, 0.37-0.74; high: HR, 0.33; 95% CI, 0.17-0.62; *P*_trend_, 8.25 × 10^-6^) decreased with the increasing of TILs, confirming the prognostic significance of TILs as a tumor biomarker (Table 1, Supplementary Tables 4, 5).

When TILs was assessed as a continuous variable, the associations between TILs and survival remained in univariable analysis, with estimated HR for each point change in intensity being 0.99 (95%CI, 0.97-1.00; *P*, 0.05) and 0.98 (95%CI, 0.96-0.99; *P*, 0.002), respectively, for the WCH cohort. These associations were validated in the TCGA cohort, with HR being 0.98 (95%CI, 0.97-0.99; *P*, 4.43 × 10^-5^) and 0.98 (95%CI, 0.97-0.99; *P*, 4.60× 10^-5^), respectively (Supplementary Table 6).

**Figure 2:**
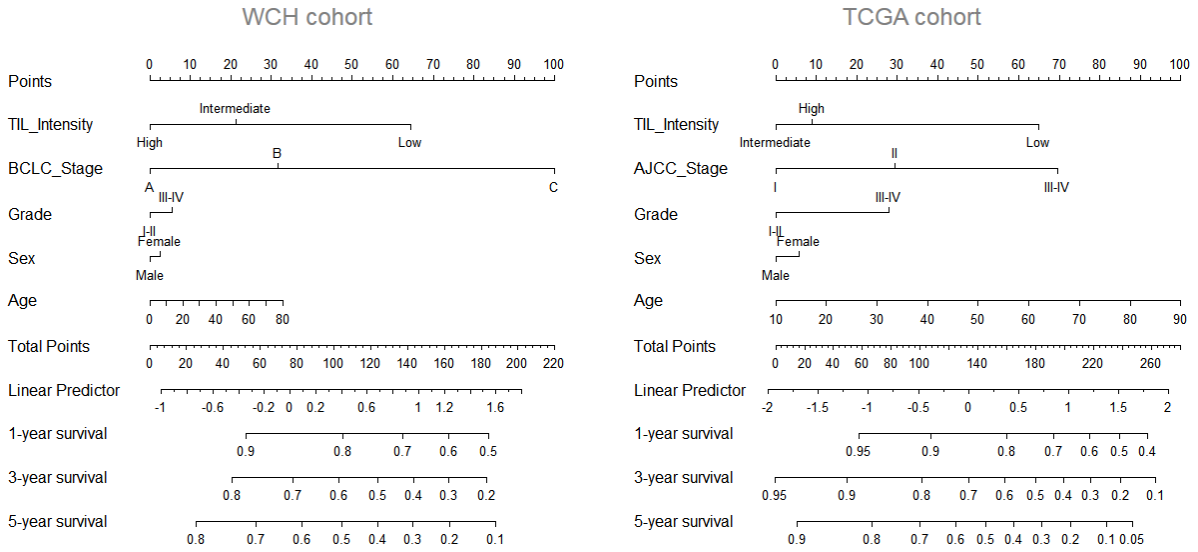
**Nomogram predicting survival possibility according to TIL intensity.** The values for each prediction factors are marked**.** From each mark, a vertical line is drawn downward to determine the points, which are subsequently added together and is marked on the Total-points line. Finally, a vertical line is followed downward to the accompanying lines indicating the 1,3 and 5 year survival possibility.

To further improve the predictive accuracy of TILs intensity by examining its performance in combination with other known prognostic factors, we constructed a nomogram incorporating TILs intensity, age, sex, tumor stage, and tumor grade for predicting the survival probability in HCC patients. As depicted in Figure 2, in both cohorts, a higher total score based on the sum of the assigned values for each prediction factor in the nomogram was associated with worse 1-year, 3-year, and 5-year OS rates. For instance, in the WCH cohort, a patient with low TIL intensity and BCLC C stage would yield a total of 164 points (64 points for low TIL intensity, and 100 points BCLC C stage), with a predicted 1, year, 3-year and 5-year OS rates of61.0%, 31.0% and 22.0%, respectively.

### HBV/HCV infection, total mutation burden or neoantigen is not associated with TILs in tumor

A recent study associated several types of oncogenic viruses with increased cytolytic activity in tumors [10]. We investigated whether HBV/HCV infection in HCC patients affected lymphocytic infiltration in tumors and para-tumor liver tissue. In para-tumor tissue, we observed a higher intensity of TILs in samples with HBV or HCV infection compared to those without infection. However, in tumor tissue, no significant difference of TILs was observed between samples with and without HBV or HCV infection (Supplementary Figure 2).

Because neoantigens derived from tumor mutations are likely to trigger an anti-tumor immune response, we tested whether neoantigens in HCC were associated with TILs in the TCGA cohort. The neoantigens data was derived from a previous study [10]. The Spearman correlation test indicated that there was no significant association between neoantigens and TILs intensity (rho = -0.13, P = 0.09). Thus, a lower neoantigens burden alone could not explain the absence of TILs in a major subset of tumors, suggesting that other mechanisms might determine the intensity of TILs in HCC (Supplementary Figure 3).

## DISCUSSION

Few studies have specifically examined the relationship between the intensity of TILs and patient mortality [11]. Most tumor marker prognostic studies on immune cells have focused on the prognostic value of immune subpopulations of the infiltrate. Distinct prognostic significance has been reported for different subpopulations. Given the functional heterogeneity of TILs, it is intriguing that the intensity of lymphocytic infiltration serving as a biomarker is of prognostic significance in HCC despite a lack of further assessment on the immune subsets of the infiltrates. Our results are consistent with the studies conducted in lung and breast cancer patients where a better prognosis was reported in patients with higher TILs [12, 13]. A possible explanation is that TILs are more likely to occur in more immunogenic tumors [14, 15]. In this setting, an overall evaluation of the immune subsets can provide a reasonably reliable estimate of the state of the local immune response. Conversely, focused evaluation of individual subsets may have limited value. For instance, the presence of a negative immune regulator (e.g., T regulatory cells) could be seen as being part of a negative feedback loop controlling the immune activation, subsequently defining tumors with preexisting anti-tumor immune response [5].

We considered two possible explanations for the variable intensity of TILs in HCC. First, we asked whether HBV/HCV infection triggered the accumulation of TILs. Indeed, both acute and chronic HBV/HCV infection has been associated with increased infiltration of immune subsets [16]. We did observe an increased intensity of TILs in the para-tumor liver tissue of patients with HBV/HCV infection, but this association was no longer valid in the tumor tissue. Our results are consistent with the study investigating the association between oncogenic viruses and cytolytic activity, where no identifiable association was observed [10]. Second, since tumor DNA mutation-derived neoantigen has been proposed to induce an anti-tumor response, [17] we tested whether predicted neoantigens or total mutation burden was associated with the intensity of TILs. Our results did not detect any significant association, suggesting the existence of other regulators for TILs.

In our study, TILs were evaluated using the methodology proposed by the International TILs Working Group. This method was initially developed for the evaluation of TILs in breast cancer, with an acceptable agreement in TILs enumeration, [5, 18] and was also used in prognosis prediction for patients with esophagus cancer, non-small-cell lung cancer, and melanoma [19–21]. We found that these criteria could be applied to HCC and could stratify patients concerning survival. We categorized the percentage of TILs as less than 10, 10-50, or more than 50. This is because these percentage breakdowns will divide the intensity of TILs into low, intermediate, or high infiltrate, as suggested by the International TILs Working Group. Our cutoff choice is also consistent with work from other groups, in which patients have been successfully stratified using these cutoffs [18, 22]. We noted that tumors with TILs more than 50%, also termed “lymphocyte-predominant cancer,” were seen in 11% of patients and had the best prognosis among the three groups.

Although our study had a relatively large sample size and a two-stage study design that included a total of 686 patients, it did have limitations. The two cohorts used in this study were from two racial groups and had distinct demographic characteristics. However, this may also mean that our findings can be generalized to these ethnicities and are not limited to one ethnic group. Another limitation is the low number (11%) of patients with TILs above 50%, which may limit the power to determine its prognostic significance.

In conclusion, we found that TILs intensity is a robust prognostic factor in patients with completely resected HCC. These results, if confirmed, suggest that clinical routine assessment of TILs could provide meaningful prognostic information in HCC.

## MATERIALS AND METHODS

### Patients and pathology materials

This study was reviewed and approved by the local ethics committee of West China Hospital (WCH); all participants provided written informed consent. The samples used in this study were unselected, nonconsecutive, primary, and historically confirmed HCCs without pretreatment that were resected at WCH (n = 316) from 2009 to 2014. Clinical and biological data, including age, sex, hepatitis B virus (HBV) infection, hepatitis C virus (HCV) infection were retrieved from computerized databases. Tumor staging was performed at the time of diagnosis following the Barcelona Clinic Liver Cancer staging system. Samples of formalin-fixed, paraffin-embedded HCC, and adjacent tissue were obtained from hepatectomy. Representative tissue areas were selected and punched from these samples and were then arranged in 1 recipient paraffin block, which was subsequently cut into 5-μm sections.

In this study, 370 HCC patients from the TCGA were also used. Level 3 RNA-sequencing data, gene mutation data, and clinical information were downloaded from the cBioPortal for Cancer Genomics (http://www.cbioportal.org), and hematoxylin and eosin (H&E) slides were accessed from the Cancer Digital Slide Archive (http://cancer.digitalslidearchive.net/). The total mutation burden and number of neoantigen were taken from previous studies [10].

The primary endpoint of the study was overall survival (OS) which was defined as the time from surgery to death from any cause. The secondary endpoint was DFS which was defined as the time from surgery to the date of disease recurrence or death from any cause.

### Pathologic assessment

**Figure 3:**
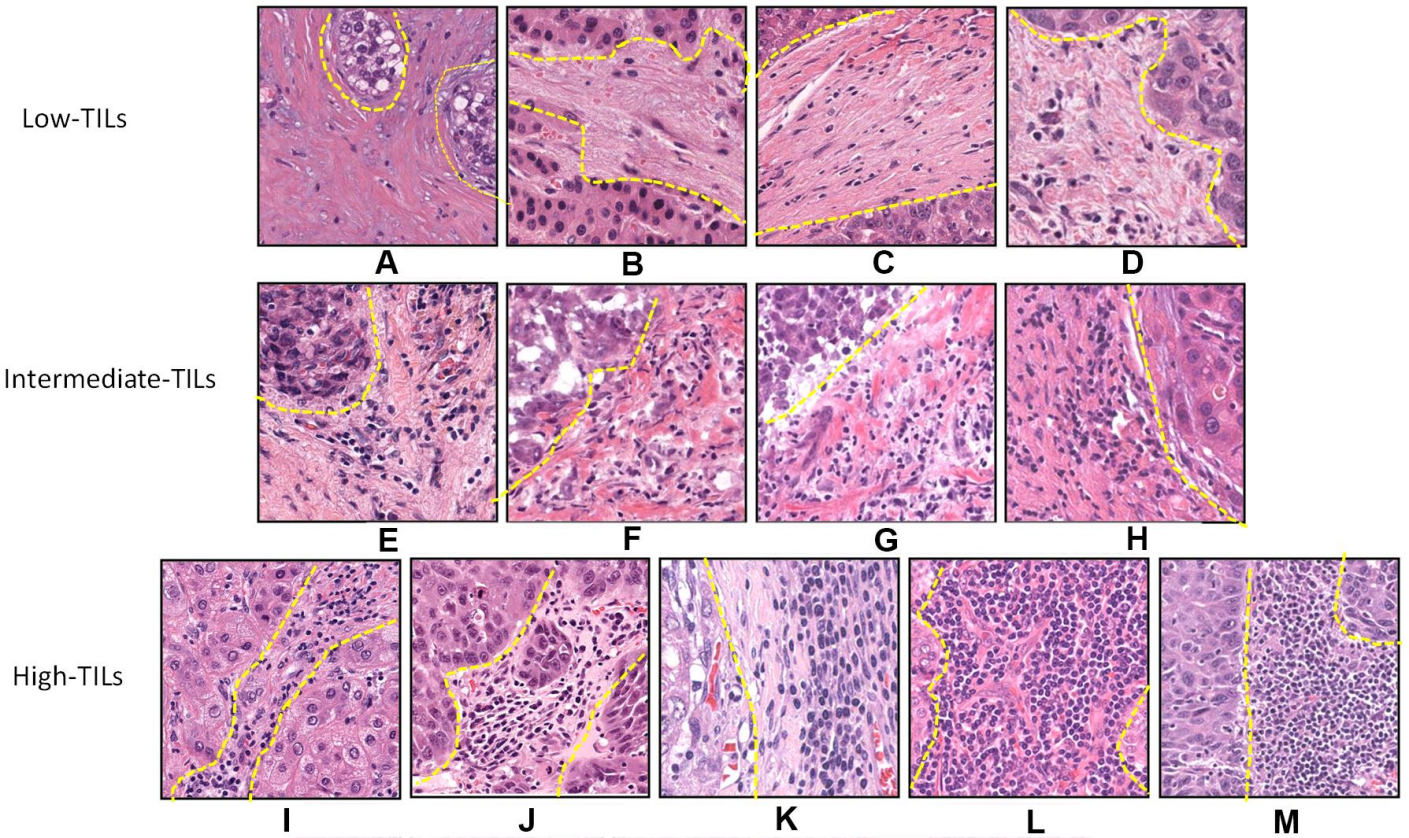
**Histopathologic examples of lymphocytic infiltration in hepatocellular carcinoma on H&E-stained tumor sections.** Example of tumors with varying intensity of stromal TILs by H&E: (**A**) 1%, (**B**) 1%, (**C**) 5%, (**D**) 5%, (**E**) 10%, (**F**) 20%, (**G**) 30%, (**H**) 40%, (**I**) 50%, (**J**) 60%, (**K**) 70%, (**L**) 80%, and (**M**) 90%.

Histopathologic evaluation of TILs intensity was jointly performed by two liver pathologists (H.W. and K.X.), who were blinded to clinical data, on H&E-stained representative sections. Boundaries of the invasive margin of the tumor were identified and only TILs inside this area were evaluated. TILs in areas with crush artifacts, necrosis, or extensive central regressive hyalinization were not scored. TILs, defined as lymphocytes infiltrated in the tumor stroma, were evaluated using the International TILs Working Group criteria [5]. According to the TILs intensity, tumors were further categorized as low (TILs < 10%), intermediate (10% ≦ TILs < 50%), and high (TILs ≧ 50%) TIL groups (Figure 3). To test analytic validity, a random subset of 100 patients from the TCGA set was selected, and TILs were assessed by an independent read. We calculated the intra-class correlation coefficient (ICC), reported as a continuous variable, with respect to consistency within observers and with respect to an agreement among observers, and we assessed the kappa statistic for TILs reported as category data. The R software package "irr" was used to estimate the ICC and kappa statistic*s.* The ICC calculated to determine internal consistency among observers was 0.63 (95% confidence interval [CI] 0.50-0.74; *P* < 0.001), and the ICC calculated to determine agreement among observers was 0.64 (95% CI 0.73-0.87; *P* < 0.001). If category cut points (<10, 10-50, ≧ 50) were used, the kappa statistic indicated moderate agreement between the two pathologists (kappa = 0.49).

Immunohistochemistry (IHC) against CD8, CD68, CD4, FOXP3, and the following quantitation was performed according to protocols which had been described in our previous study [23].

### Statistical analysis

All statistical tests were two-sided, and *P* values less than 0.05 were considered statistically significant. Differences between groups were compared by the χ^2^ test or Fisher’s exact test for categorical variables and by Student’s t-test for continuous variables. The Spearman correlation test was used to determine the extent of correlation between TILs and the immune-related variables. The survival rates of the high-, intermediate-, and low-TILs groups were estimated using the Kaplan-Meier method. OS and DFS were assessed using the log-rank test. The hazard ratios (HRs) and 95% CIs for TILs were estimated through univariable and multivariable Cox regression model while adjusting for known histopathologic and demographic prognostic factors (age, sex, tumor stage, and grade). Cases with missing data items were also included in the analysis by categorizing them as “missing”. Statistical analysis was performed using R software (R 3.4.1). REMARK criteria were followed in this study [24].

## Supplementary Material

Supplementary Figures

Supplementary Tables
