# Supplementary material for: The density of tumor-infiltrating lymphocytes and prognosis in resectable hepatocellular carcinoma: a two-phase study
Source: Aging (Albany NY). 2021 Mar 19;13(7):9665–78. doi: 10.18632/aging.202710 (PMC8064144; doi:10.18632/aging.202710)
Supplement: Supplementary Figures [file aging-13-202710-s001.pdf]

SUPPLEMENTARY FIGURES

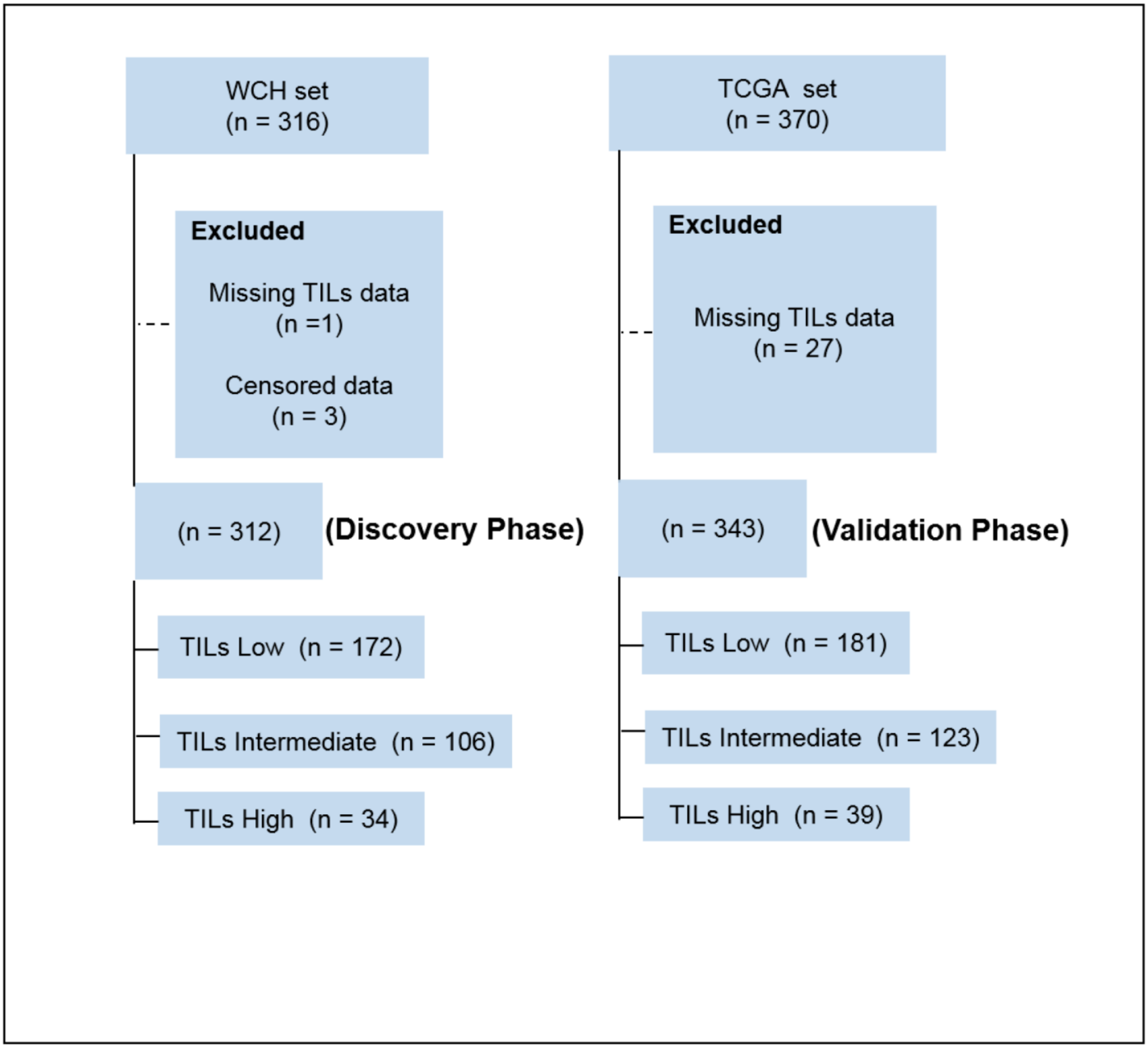

Supplementary Figure 1. Flow diagram of HCC tissue samples and TCGA dataset used in this study. Patients with missing TILs data correspond to patients with blocks of insufficient performance for quantitative evaluation, mostly due to necrosis tissue or tissue section separation. Censored data indicated those without definite death time. WCH, West China Hospital; TCGA, The Cancer Genome Atlas.

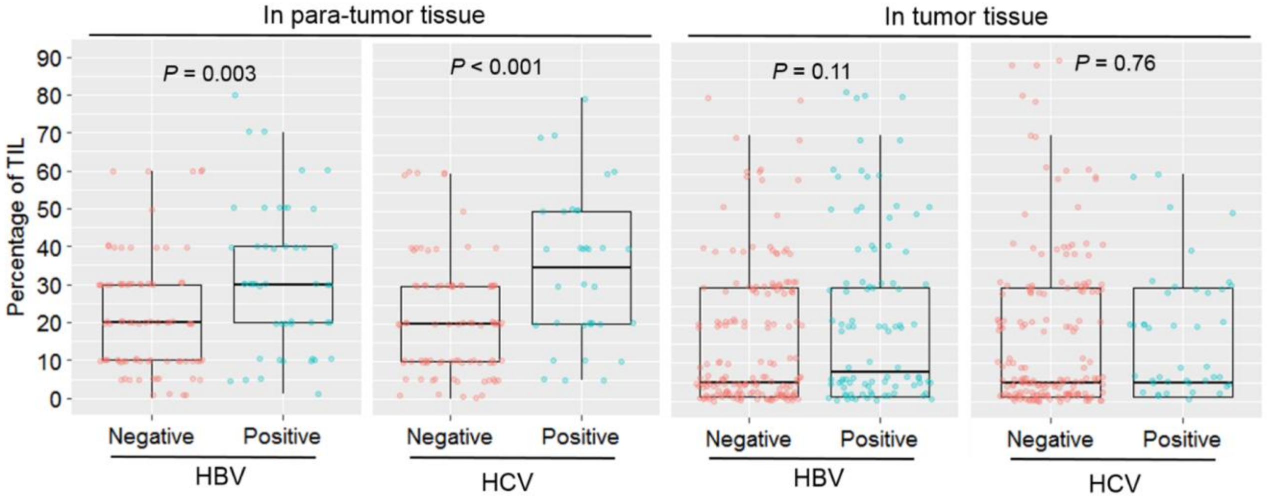

Supplementary Figure 2. Box plots showing TILs intensity in para-tumor and tumor samples with and without HBV/HCV infection.

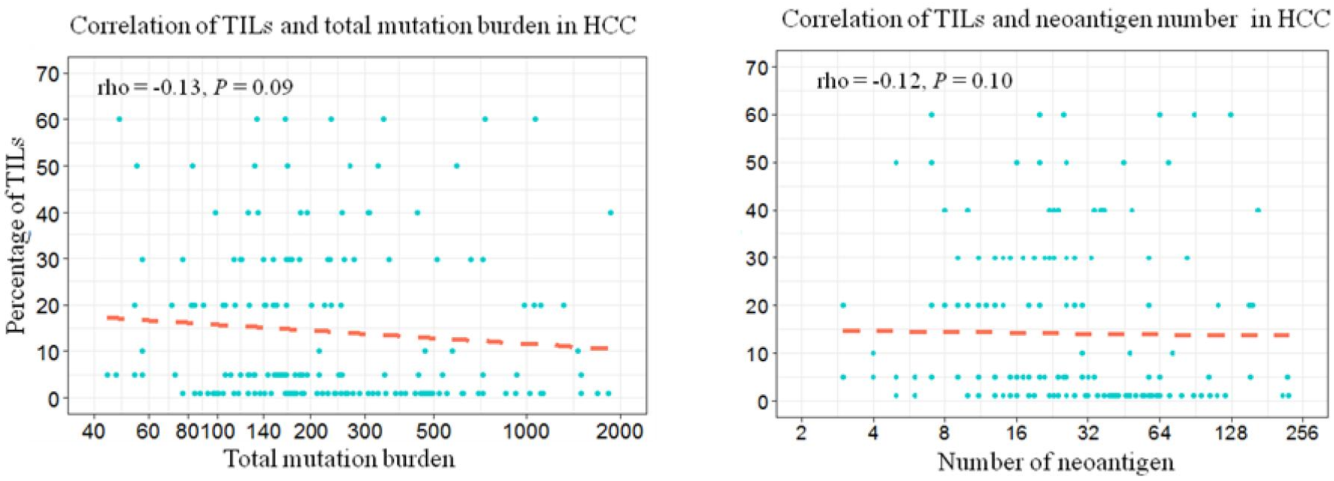

Supplementary Figure 3. Local regression curves show that there is no significant correlation of TILs intensity with total mutation burden and number of predicted neoantigens.
