# Supplementary material for: The density of tumor-infiltrating lymphocytes and prognosis in resectable hepatocellular carcinoma: a two-phase study
Source: Aging (Albany NY). 2021 Mar 19;13(7):9665–78. doi: 10.18632/aging.202710 (PMC8064144; doi:10.18632/aging.202710)
Supplement: Supplementary Tables [file aging-13-202710-s002.pdf]

SUPPLEMENTARY TABLES

Supplementary Table 1. Characteristics of patients in the West China Hospital (WCH) and The Cancer Genome Atlas (TCGA) sets.

Characteristic	WCH set (n=316)	TCGA set (n=370)	P
Sex			<0.001
Male	258 (81)	249 (66)	
Female	58 (19)	121 (31)	
Age, years			<0.001
Mean	51.28	59.35	
≤40	55 (19)	32 (8)	
40-60	181 (54)	145 (36)	
≥60	80 (27)	193 (52)	
Race			<0.001
Asian	316 (100)	157 (45)	
White	0	184 (47)	
Others	0	29 (4)	
Risk factor			<0.001
HBV	214 (99)	104 (30)	
HCV	3 (1)	56 (16)	
Alcohol	0	117 (33)	
Nonalcoholic steatohepatitis	0	20 (6)	
Other	0	11 (3)	
No history of primary risk factors	0	91 (26)	
NA	99	19	
Serum AFP level, ng/ml			0.007
>20	181 (58)	130 (47)	
≤20	131 (42)	147 (53)	
NA	4	93	
No. of tumors			
1	227 (82)		
>1	51 (18)		
NA	38	370	
Mean tumor size (range), cm	5.98 (0.3-19)	NA	
Vascular invasion			0.672
Yes	97 (31)	108 (34)	
No	219 (69)	206 (66)	
NA		56	
Tumor differentiation			0.084
I	7 (2)	55 (14)	
II	160 (55)	177 (47)	
III/IV	122 (42)	133 (35)	
NA	27	5	
Ishak score			<0.001
0	70 (25)	74 (35)	
1-2	10 (4)	31 (15)	
3-4	59 (21)	28 (13)	
5-6	142 (50)	78 (37)	
NA	35	159	

Percentage of TILs		
1	122 (39)	111 (32)
5	53 (17)	70 (21)
10	51 (16)	15 (4)
20	39 (12)	41 (12)
30	14 (4)	47 (14)
40	2 (1)	20 (6)
50	13 (4)	12 (3)
60	9 (3)	13 (4)
70	3 (1)	4 (1)
80	5 (2)	6 (2)
90	4 (1)	4 (1)
NA	1	27
No. of deaths	99 (33)	130 (34)
Median follow-up time (range), months	34 (1-93)	19 (0-120)

Note: Data are no. of patients (%) unless otherwise indicated.

Abbreviations: HBV, hepatitis B virus; HCV, hepatitis C virus; AFP, alpha fetoprotein; NA, not available.

Supplementary Table 2. Expression of immune-related markers by the intensity of TILs.

	TILs-low (n=175)	TILs-high (n=34)	*P*
CD8			
Low	100	8	4.59×10^{-4}
High	74	25	
CD4			
Low	62	11	0.21
High	56	17	
CD68			
Low	95	14	0.21
High	80	19	
OX40			
Low	110	15	0.04
High	65	19	
PD-1			
Low	160	21	8.62×10^{-6}
High	15	12	
PD-L1_tumor			
Negative	145	26	0.23
Positive	18	6	
PD-L1_immume cell			
Negative	141	21	3.97×10^{-3}
Positive	22	11	

Supplementary Table 3. Mortality risks by the intensity of TILs for overall survival and disease-free survival in univariable analysis.

Group	TILs intensity	Overall survival			Disease-free survival		
		No. of cases/total	HR	*P*	No. of cases/total	HR	*P*
			**Discovery phase (WCH set)**				
Low	TILs < 10%	67/172	1 (reference)		80/103	1 (reference)	
Intermediate	10% ≦ TILs <50%	24/106	0.54 (0.34-0.86)	0.01	21/58	0.34 (0.21-0.56)	1.56×10^{-5}
High	TILs ≧ 50%	5/34	0.33 (0.13-0.83)	0.02	5/22	0.21 (0.09-0.52)	7.86×10^{-4}
P **for trend**				1.14×10^{-3}			6.50×10^{-7}
			**Validation phase (TCGA set)**				
Low	TILs < 10%	81/181	1 (reference)		100/154	1 (reference)	
Intermediate	10% ≦ TILs <50%	30/123	0.44 (0.29-0.67)	1.45×10^{-4}	50/111	0.53(0.38-0.74)	2.78×10^{-4}
High	TILs ≧ 50%	8/39	0.32 (0.16-0.67)	2.40×10^{-3}	11/33	0.30 (0.16-0.55)	6.09×10^{-4}
P **for trend**				1.60×10^{-6}			1.38×10^{-6}

Supplementary Table 4. Mortality risks by the intensity of TILs, tumor stage, and tumor grade for overall survival and disease-free survival in the WCH cohort.

	Overall survival			Disease-free survival		
	No. of cases/total	HR	P	No. of cases/total	HR	P
TILs intensity						
Low	67/172	1 (reference)		80/103	1 (reference)	
Intermediate	24/106	0.58 (0.36-0.93)	0.02	21/58	0.35 (0.22-0.58)	4.59 × 10-5
High	5/34	0.37 (0.15-0.93)	0.04	5/22	0.23 (0.09-0.58)	1.83 × 10-3
Stage (BCLC)						
A	61/218	1 (reference)		63/121	1 (reference)	
B	19/47	1.57 (0.92-2.69)	0.10	21/31	1.52 (0.92-2.52)	0.11
C	10/13	3.92 (1.97-7.81)	1.03 ×10-4	12/13	3.29 (1.70-6.37)	4.18 × 10-4
Tumor grade						
I-II	48/167	1 (reference)		56/100	1 (reference)	
III-IV	44/122	1.16 (0.76-1.77)	0.49	45/74	1.10 (0.74-1.65)	0.63

Supplementary Table 5. Mortality risks by the intensity of TILs, tumor stage, and tumor grade for overall survival and disease-free survival in the TCGA cohort.

	Overall survival			Disease-free survival		
	No. of Cases/Total	HR	P	No. of Cases/Total	HR	P
TILs intensity						
Low	81/181			100/154		
Intermediate	30/123	0.42 (0.28-0.66)	1.13×10^{-4}	50/111	0.52(0.37-0.74)	2.78×10^{-4}
High	8/39	0.41 (0.19-0.86)	0.02	11/33	0.33 (0.17-0.62)	6.09×10^{-4}
Stage (AJCC)						
I	42/168			61/147		
II	26/84	1.57(0.94-2.61)	0.08	43/71	2.11(1.40-3.17)	3.37×10^{-4}
III-IV	44/84	2.74(1.75-4.27)	9.56×10^{-6}	49/70	2.55(1.73-3.78)	2.73×10^{-6}
Tumor grade						
I-II	75/234			99/192		
III-IV	46/130	1.57 (1.05-2.33)	0.03	63/112	1.33 (0.96-1.86)	0.09

Supplementary Table 6. Mortality risks with each point change in TILs intensity in univariable analysis.

	Overall survival		Disease-free survival	
	HR	*P*	HR	*P*
WCH cohort	0.99 (0.97-1.00)	0.05	0.98 (0.96-0.99)	0.002
TCGA cohort	0.98 (0.97-0.99)	4.43×10^{-5}	0.98(0.97-0.99)	4.60×10^{-5}
